# Survival advantage observed with the use of metformin in patients with type II diabetes and colorectal cancer

**DOI:** 10.1038/bjc.2012.71

**Published:** 2012-03-15

**Authors:** C R Garrett, H M Hassabo, N A Bhadkamkar, S Wen, V Baladandayuthapani, B K Kee, C Eng, M M Hassan

**Affiliations:** 1 Department of Gastrointestinal Medical Oncology, MD Anderson Cancer Center, Unit 426, 1515 Holcombe Boulevard, Houston, TX, 77030-4009, USA; 2 Department of General Oncology, MD Anderson Cancer Center, Unit 462, 1515 Holcombe Boulevard, Houston, TX 77030-4009, USA; 3 Department of Biostatistics, MD Anderson Cancer Center, Unit 1411, 1515 Holcombe Boulevard, Houston, TX, 77030-4009, USA

**Keywords:** colorectal cancer, metformin, diabetes, overall survival, retrospective review

## Abstract

**Background:**

Patients with type II diabetes mellitus (DM) have an increased risk of adenomatous colorectal (CRC) polyps and CRC cancer. The use of the anti-hyperglycemic agent metformin is associated with a reduced incidence of cancer-related deaths.

**Methods:**

We retrospectively evaluated the medical records of 4758 patients seen at a single institution and determined that 424 patients were identified by their physicians as having type II DM and CRC cancer. Data were subsequently acquired determining the subject's age, body mass index (BMI), and disease date of diagnosis, stage, site of cancer, treatment, and survival.

**Results:**

Patients with type II DM and CRC cancer treated with metformin as one of their diabetic medications had a survival of 76.9 months (95% CI=61.4–102.4) as compared with 56.9 months in those patients not treated with metformin (95% CI=44.8–68.8), *P*=0.048. By using a multivariable Cox regression model adjusted for age, sex, race, BMI, and initial stage of disease, we demonstrated that type II diabetic patients treated with metformin had a 30% improvement in overall survival (OS) when compared with diabetic patients treated with other diabetic agents.

**Conclusion:**

Colorectal cancer patients with DM treated with metformin as part of their diabetic therapy appear to have a superior OS.

Diabetes mellitus (DM), specifically type II DM, is associated with an increased risk of cancer, particularly pancreas, liver, endometrium, breast, bladder, and colorectal (CRC; Vigneri *et al*, 2009). This increased risk observed is independent of age and body mass index (BMI). A meta-analysis of 15 studies involving a total of 2 593 935 participants demonstrated that the relative risk of CRC in type II DM patients compared with those without the disease was 1.30 (95% CI=1.2–1.4; Larsson *et al*, 2005). Type II DM patients with poor glycemic control, as measured by glycosylated haemoglobin (HbA1c), have a greater prevalence of right-sided adenomatous polyps, a greater number of polyps, and a greater number of adenomatous polyps (Siddiqui *et al*, 2008). The cause for the increased risk of cancer in type II DM patients is not clearly understood but may be related to dietary-induced elevated insulin (Venkateswaran *et al*, 2007) and elevated insulin-like growth factor-I levels (Ma *et al*, 1999). Insulin is a humeral growth factor for a variety of cancer cells *in vitro*, and a variety of therapies targeting the insulin-like growth factor receptor are undergoing evaluation as potential anti-cancer treatment (Maki, 2010; Golan and Javle, 2011).

Metformin is a biguanide widely used to decrease plasma glucose levels by increasing intracellular glucose uptake (Bailey and Turner, 1996). Type II DM population studies demonstrate that patients who use only metformin for management of their DM have a lower cancer risk (1.08, CI=0.96–1.21) compared with those treated with metformin plus a sulphonylurea (1.36, 95% CI=1.19–1.54), *vs* those on insulin-based regimes (1.42, 95% CI=1.27–1.60; Currie *et al*, 2009). Metformin has been associated with a lower cancer mortality in type II DM as compared with its non-use (Landman *et al*, 2010). In Korean patients with CRC, a recent report (Lee *et al*, 2011) indicated that metformin use was associated with lower risk of overall mortality, especially patients with stage III. The current study aimed to assess such association in US patients after controlling for confounding effect of several factors related to CRC survival.

## Materials and Methods

Under an Institutional Review Board approved protocol (designated DR09-1719) the electronic records of 4758 patients with a diagnostic code of colon cancer or rectal cancer seen at a single institution (MD Anderson) from 1 January 2004 to 31 December 2008 were reviewed. All patients were diagnosed with pathologically confirmed CRC and were evaluated at their baseline visit to MD Anderson for appropriate staging according to Tumour/Node/Metastatic (TNM) scoring systems staging. Structured data collection sheet was developed to retrieve epidemiological and clinical factors. A manual retrospective review was conducted for all patients to identify those with prior history of DM.

Of these 4758 patients, 424 were identified by their cancer physician at the time of their initial consultation as having type II DM treated with diet, medication, or insulin (or a combination of these therapies); patients identified as having type I DM were excluded from this analysis. Only patients who resided in the United States were included in this study. Patients were identified by review of outside records as having type II DM; additional confirmatory testing was not routinely performed. Follow-up information for survival was available in 397 patients. Date of diagnosis, patient's age, height, weight, and BMI at the time of diagnosis were recorded. Body mass index were stratified into ‘underweight’ (BMI<18.5), ‘normal’ (BMI range: 18.5–24.9), ‘overweight’ (BMI range: 25.0–29.9), and ‘obese’ (BMI⩾30.0). A total of 13 patients referred to MD Anderson without baseline images including computed tomography scan, magnetic resonance imaging and were excluded from the analysis. Tumour-specific informations, including size, pathologic stage, lymphovascular invasion, as well as adjuvant and metastatic cancer therapies, were abstracted from the medical record. Diabetic medications, glycosylated haemoglobin (HbA1c), and anti-cholesterol medications were also noted. The pathologic response rate following chemoradiation therapy for rectal cancers and perioperative chemotherapy for hepatic metastases undergoing surgical resection were noted.

### Statistical considerations

All clinical and epidemiological data were merged and analysed with use of STATA software (STATA Corp., College Station, TX, USA). Overall survival (OS) was defined as the time between dates of CRC diagnosis and death or end of follow-up (censored observations). Median survival was estimated by using the Kaplan–Meier product-limit method, and significant differences in survival times among CRC with and without DM and different DM treatment were determined by using the log-rank test. Hazard ratios (HRs) and 95% CIs were calculated by using Cox proportional hazard models with a backward stepwise selection procedure, considering the clinical co-variates of CRC. Univariate analyses were conducted with *χ*^2^ or Fisher exact tests for categorical variables and the Kruskal–Wallis test for continuous variables.

## Results

**Figure 1:**
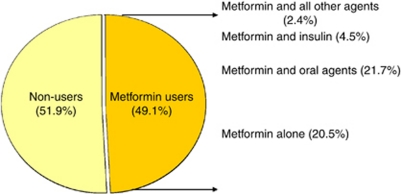
Distribution of metformin intake in patients with CRC.

**Figure 2:**
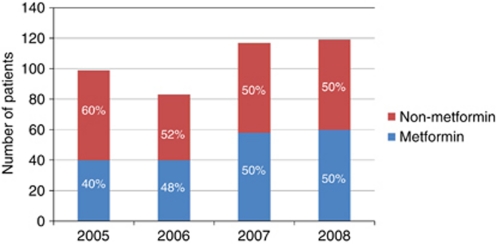
Metformin usage (by calendar year).

Among 4758 CRC patients the prevalence of type II DM was 8.9% (424 patients); the overall mean age (±s.d.) was 62.7 years (±10.2). Men to women ration were approximately 2 : 1. Majority of patients were white, which was consistent with referral pattern of MD Anderson Cancer Center. Table 1 showed the demographic and clinical features of CRC patients with type II DM stratified by metformin intake. We found no significant difference between both groups. International patients, for whom follow-up data would not be available, were not included in this analysis. The types of anti-diabetic medications administered to the patients are demonstrated in Figure 1. There was a slight preponderance of patients not receiving metformin in 2005, with relatively equal distribution in the years 2006–2008 (see Figure 2). HbA1c was performed at the time of the initial consultation in 118 patients (28%); median HbA1c was 7.2% (range 4.9–12.9%). Aspirin was used at the time of original evaluation at MD Anderson in 119 of 424 patients (28.1%); there was no significant difference in aspirin use in CRC diabetic patients treated with and without metformin, *P*=0.3 (Table 2). Anti-cholesterol therapy at the time of the patient's first evaluation was 43% and was slightly higher in the non-metformin group when compared with the metformin group (47% *vs* 39%).

**Figure 3:**
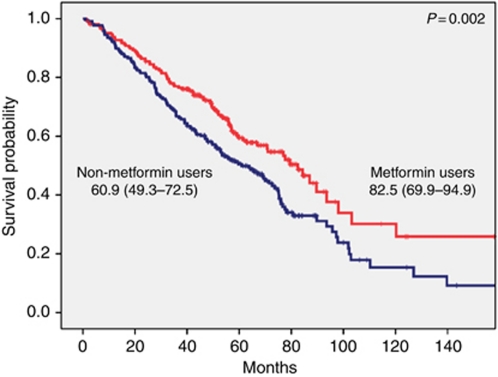
Figure 2: Overall median survival (95% CI) in months with comparison between metformin and non-metformin users in all patients with CRC.

**Figure 4:**
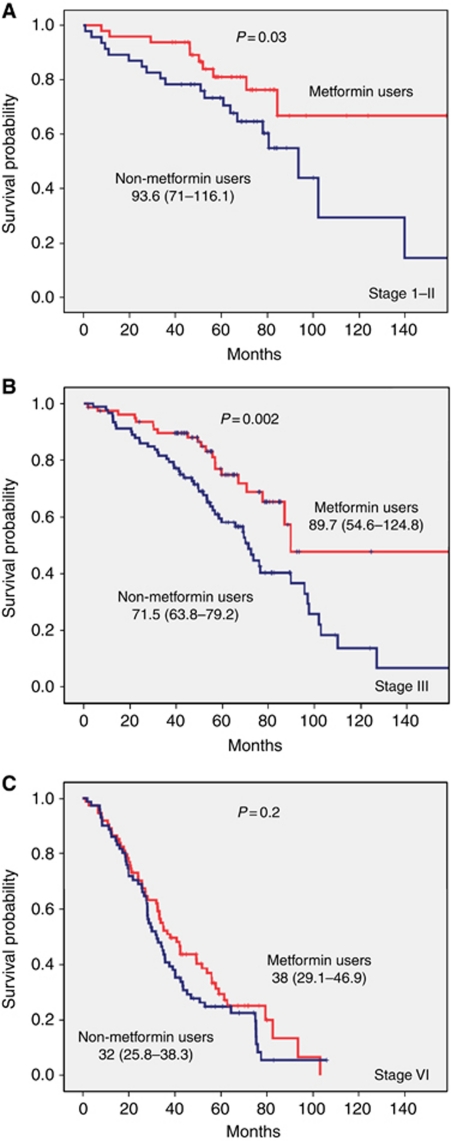
Overall median survival (95% CI) in months with comparison between metformin and non-metformin users stratified by TNM staging in all patients with CRC: **A** (stage I–II), **B** (stage III), **C** (stage IV).

For survival analysis, death was confirmed for 194 CRC patients (45.8%); the median OS was 70.7 months (95% CI= 62.3–79.1). Patients with type II DM and CRC treated with metformin had significantly longer OS of 82.5 months (95% CI= 69.9–94.9) as compared with 60.9 months in patients not treated with metformin (95% CI=49.3–72.5), *P*=0.002 (Figure 3). The significant difference between metformin- and non-metformin-treated patients was observed in patients with TNM stage I–II and in patients with TNM-stage III (Figure 4). The estimated median OS times (95% CI) were 89.7 (54.6–124.8) compared with 71.5 (63.8–79.2), *P*=0.002. However, survival of metformin-treated patients with stage I–II did not reach the median. No significant difference was observed in advanced stage CRC patients (Stage IV). There was a non-statistical significant trend towards a higher complete and minor pathologic response rate (⩽10% residual tumour) in type II DM patients with rectal cancer receiving neoadjuvant chemoradiation who were treated with metformin *vs* those who were not (14/19, 74% *vs* 9/19, 47%, *P*=0.09). Adjusting for age, sex, race, BMI, aspirin usage, and initial stage of disease indicated that type II DM patients with CRC treated with metformin had a 40% improvement in OS when compared with type II DM patients treated with other anti-diabetic agents. The estimated HR (95% CI) was 0.6 (0.5–0.8). Including BMI as continuous or categorical variable did not meaningfully change the observed lack of predictive role of baseline obesity on the OS of CRC patients.

## Discussion

Of 424 patients with CRC cancer, we found a prevalence of type II DM of 8.9% this is similar to other reports, which noted a prevalence of 11% (Lee *et al*, 2011), 7.9% (Meyerhardt *et al*, 2003), and 9.6% (van de Poll-Franse *et al*, 2007). In this retrospective analysis, the use of metformin in type II DM patients with CRC was associated with an improved OS. This retrospective analysis is limited by the fact that data were not present regarding the date of onset of DM as well as the duration of exposure of the patient to metformin; a carefully controlled prospective study would be required to confirm these preliminary results. The median 5-year OS of 50% is somewhat lower than the 63% 5-year survival reported from Surveillance, Epidemiology, and End Results data 2004–2008 (SEER, 2011) and likely reflects the referral bias (patients with more advanced stage and with refractory disease being referred to a quaternary cancer centre). Although subject to all of the limitations of a retrospective analysis, the findings are consistent with data from other solid tumours including CRC cancer. Metformin usage has been shown to reduce cancer-specific and overall mortality in patients with type II DM on metformin compared with diabetic patients not taking metformin (Lee *et al*, 2011). The use of metformin in diabetic patients has been associated with a decreased risk of pancreas cancer (Li *et al*, 2009), hepatocellular cancer (Hassan *et al*, 2010), ovarian cancer (Bodmer *et al*, 2011), and breast cancer (Decensi *et al*, 2010). Metformin use has been associated with an improved survival in patients with pancreatic cancer (Sadeghi *et al*, 2011) and lung cancer (Gagnon *et al*, 2009). In patients with CRC cancer treated with chemotherapy, metformin was associated with an improved survival rate (Bansal *et al*, 2011). However, a retrospective review demonstrated that metformin did not significantly impact the outcome of survival of patients when used in the adjuvant setting in triple receptor-negative breast cancer (Bayraktar *et al*, 2011). In patients with prostate cancer, for which the association between increased risk and DM is less certain, an improved survival was not observed with the use of metformin (Azoulay *et al*, 2011).

The mechanism whereby metformin reduces glucose levels in diabetic patients is not entirely clear. However, it has been recently demonstrated to impair mitochondrial ATP production leading to the activation of liver kinase B1 (LKB1)-5′ AMP-activated protein kinase (AMPK; El-Mir *et al*, 2000; Hawley *et al*, 2010). This leads to a decrease in energy-consuming processes in order to restore ATP levels (Kahn *et al*, 2005). Metformin LKB1 activation in human cancer cell lines *in vitro* is associated with inhibition of the mammalian target of rapamycine (mTOR), with subsequent reduced cellular proliferation (Shaw *et al*, 2004a, 2004b; Dowling *et al*, 2006; Zakikhani *et al*, 2006). Thus, in cancer cells metformin inhibited insulin-stimulated mTOR activation and proliferation in an AMPK-dependent manner (Engelman and Cantley, 2010; Pollak, 2010). *In vitro* metformin has also been demonstrated to induce apoptosis in human ovarian cancer cell lines by directly activating caspases 3/7, downregulating Bcl-2 and Bcl-xl expression, whereas upregulating Bax and Bad expression (Yasmeen *et al*, 2011). Metformin may also potentially have an immunomodulatory role. Work is ongoing to determine the likely most relevant anti-cancer mechanism of metformin in diabetic patients.

Based upon these epidemiologic observations, as well as significant preclinical data suggesting a cancer prevention and therapeutic anti-cancer role for metformin in certain malignancies, a National Cancer Institute of Canada prospective randomised trial in early breast cancer has been initiated (Parulekar *et al*, 2011). Future prospective trials will be required in type II DM patients with CRC to confirm these retrospective findings of a survival benefit associated with the use of metformin in type II DM patients with CRC.

## Figures and Tables

**Table 1:** Characteristics of type 2 DM patients with CRC by their intake of metformin

		**Metformin users**		**Non-metformin users**		
**Variable**	**Variable label**	***N*=208**	**%**	***N*=216**	**%**	***P*-value**
Sex	Male	141	67.8	142	65.7	0.7
	Female	67	32.2	74	34.3	
						
Age (years)	⩽50	21	10.1	25	11.6	0.7
	51–59	70	33.7	64	29.6	
	60–69	74	35.6	71	32.9	
	>70	43	20.7	56	25.9	
						
Race	White	142	68.3	145	67.1	0.7
	African Americans	27	13	30	13.9	
	Hispanics	26	12.5	32	14.8	
	Asians	13	6.3	9	4.2	
						
BMI Status	Underweight/normal	45	21.6	44	20.4	0.9
	Overweight	62	29.8	67	31	
	Obese	101	48.6	105	48.6	
						
Aspirin intake	No	145	69.7	160	74.1	0.3
	Yes	63	30.3	56	25.9	
						
TNM staging^a^	Stage I–II	48	23.8	46	22	0.6
	Stage III	80	36.9	92	44	
	Stage IV	74	36.6	71	34	
						
Cancer site^a^	Rectum	58	27.9	71	32.9	0.9
	Recto-sigmoid	19	9.1	17	7.9	
	Sigmoid	48	23.1	45	20.8	
	Ascending	47	22.6	40	18.5	
	Transverse	19	9.1	22	10.2	
	Descending	8	3.8	11	5.1	
	Synchronous	3	1.4	3	1.4	

Abbeviations: BMI=body mass index; CRC=colorectal; DM=diabetes; TNM=tumour/node/metastatic.

a Thirteen patients (six from metformin users and seven from non-metformin users) without baseline imaging (computed tomography or magnetic resonance imaging).

**Table 2:** Survival prediction of metformin intake: adjusted HR using Cox regression^a^

**Variable**	**Variable label**	**HR (95% CI)**	***P*-value**
Sex	Male	1 (Reference)	
	Female	0.8 (0.6–1.1)	0.07
			
Age categories	⩽50	1 (Reference)	
	51–60	0.8 (0.5–1.2)	0.5
	61–70	1.1 (0.7–1.7)	0.5
	>70	1.2 (0.8–1.9)	0.2
			
Race	Non-white	1 (Reference)	
	White	0.9 (0.7–1.3)	0.6
			
BMI		1 (0.9–1.1)	0.9
Aspirin intake	No	1 (Reference)	
	Yes	0.8 (0.6–1.1)	0.3
			
Staging category	Stage I–II	1 (Reference)	
	Stage III	1.6 (1.02–2.4)	**0.04**
	Stage IV	5.6 (3.7–8.4)	**0.0001**
			
Metformin intake	No	1 (Reference)	
	Yes	0.6 (0.5–0.8)	**0.001**

Abbreviations: BMI=body mass index; CI=confidence iterval; HR=hazard ratio.

a Adjusted for age, sex, race, BMI, aspirin usage, and tumour/node/metastatic staging.

The bold values are statistically significant.
